# Design and evaluation of the pneumatic leg prosthesis ERiK to assist elderly amputees with sit-down and stand-up movements

**DOI:** 10.1017/wtc.2023.8

**Published:** 2023-05-26

**Authors:** Heike Vallery, Frederik Lachmann, Simon van der Helm, Andrew Pennycott, Gerwin Smit

**Affiliations:** 1 Faculty of Mechanical, Maritime and Materials Engineering, TU Delft, Delft, The Netherlands; 2 Department of Rehabilitation Medicine, Erasmus MC, Rotterdam, The Netherlands

**Keywords:** Knee exoprosthesis, sit-to-stand, lightweight pneumatic actuator, transfemoral amputation

## Abstract

Standing up using one leg is a challenging task for those with a transfemoral amputation, particularly for elderly users with a low activity level. Active prostheses are generally not accessible to this group and available passive prostheses do not support standing up. This article presents the design and evaluation of the “Energy Restoring Intelligent Knee” (ERiK), which stores energy during sit-down in a pneumatic cylinder and returns it during stand-up. We hypothesized that the system would reduce the time needed to perform transitions and also enable higher load sharing by the prosthetic leg. However, the results of an experimental study with seven participants with transfemoral amputation contradict these hypotheses: the participants could neither move faster nor make more use of the prosthetic leg to share their body weight during transitions. We observed that a major obstacle to the useful functionality of the leg was the absence of ankle dorsiflexion – the foot tended to slip during stand-up initiation, such that only low pre-pressures and therefore support levels could be set. The rather binary action of the pneumatics also complicated movement initiation. The lessons learned from this study may be helpful to those seeking to create better designs in the future.

## 1. Introduction

With 60 stand-up (SU) transitions per day, the sit-to-stand movement is a frequently exercised activity (Dall and Kerr, 2010). Standing up involves the entire body and can be more demanding than other daily routines. It requires significant torque and a greater range of motion at the knee and the ankle joints (Kralj et al., 1990; Schenkman et al., 1990; Janssen et al., 2002; Lindemann et al., 2003; Galli et al., 2008) in comparison to stair climbing or walking (Berger et al., 1988; Hodge et al., 1989). Indeed, Riley et al. (1991) consider it “the most mechanically demanding functional task routinely undertaken during daily activities.”

Especially elderly and persons with impairments such as transfemoral amputations have problems in standing up (Dawson et al., 1987; Burger et al., 2005). After an amputation, less than half of elderly people achieve a household activity level (Fortington et al., 2012). In the Netherlands, there are currently 9,680 people (6,530 male, 3,150 female) registered who use a prosthetic foot or leg out of the 16 million people registered for health insurance. Of this group, 5,200 (53%) are aged 65 or older (GIP, 2021).

A study from the Northern Netherlands showed that 34% of the lower limb amputations were transfemoral amputations (Fortington et al., 2013). The study also showed that lower limb amputation incidence had remained quite constant over a period of 12 years, despite improvements in healthcare.

Regaining the capability to independently perform daily routine motions greatly improves the quality of daily life for amputees. One of the most effective ways to regain mobility is the use of a transfemoral prosthesis (Inoue et al., 2016). However, users of transfemoral prostheses without active support mechanisms often struggle to stand up independently and they often depend on external assistance (Varol et al., 2009). In comparison to able-bodied persons, the independent SU transition for transfemoral amputees requires approximately twice the torque from the sound knee joint (Burger et al., 2005). The inability to stand up independently may lead to institutionalization (Schultz et al., 1992).

In order to reduce prosthesis weight and energy requirements, it is attractive to look at energy harvesting from human movement so that the energy can be released at a later point in time. This has been done mainly for gait, as in Arnout et al. (2012), but it is also particularly intuitive for stand-up and sit-down (SD) movements. With few exceptions, such as Sup et al. (2008), current active prostheses are mostly electric, such as Flynn et al. (2018), Lenzi et al. (2018), and Azocar et al. (2020). If electric motors are used as generators to recuperate energy, the mechanical energy is transformed into electrical energy, stored in a battery or capacitor, and later recuperated. Such energy transformation inevitably incurs losses. Furthermore, most research focuses on walking, and there is less emphasis on designs that only assist standing-up and sitting-down movements.

Here, we investigate a very simple mechanical concept that stores and releases mechanical energy directly – without transformation – during stand-to-sit and sit-to-stand motions. Specifically, we designed a pneumatic prosthetic knee that regenerates and stores the energy of sitting down and uses this energy to deliver support during standing up. During walking, the knee joint is locked in a fully extended state. The prosthesis is intended for users with a low activity level who perform mainly in-house walking and prefer the security of a locked knee. In the remainder of this article, we explain the design and technical evaluation of the system and present the results of a pilot study with individuals with transfemoral amputation.

## 2. Design of the Energy Restoring Intelligent Knee prosthesis

### 2.1. General concept and pneumatic scheme

**Figure 1.**
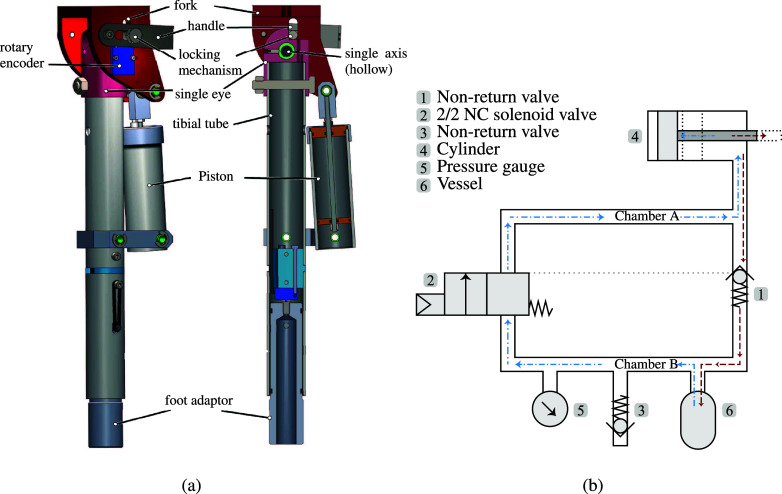
Design of the Energy Restoring Intelligent Knee (ERiK) prosthesis. (a) CAD drawings with assembled view and cross-section view in the sagittal plane. ERiK’s main element is a pneumatic cylinder that acts like a gas spring. When sitting down, the air is pressurized by the piston in the upper chamber. When standing up, the air in the upper chamber is decompressed. (b) Pneumatic scheme: When sitting down, the air is pressurized by the piston (4) and flows through the nonreturn valve (1) to the accumulator vessel (6). When standing up, the solenoid valve (2) opens, and the air flows back to the cylinder, thus exerting a force on the piston.

**Figure 2.**
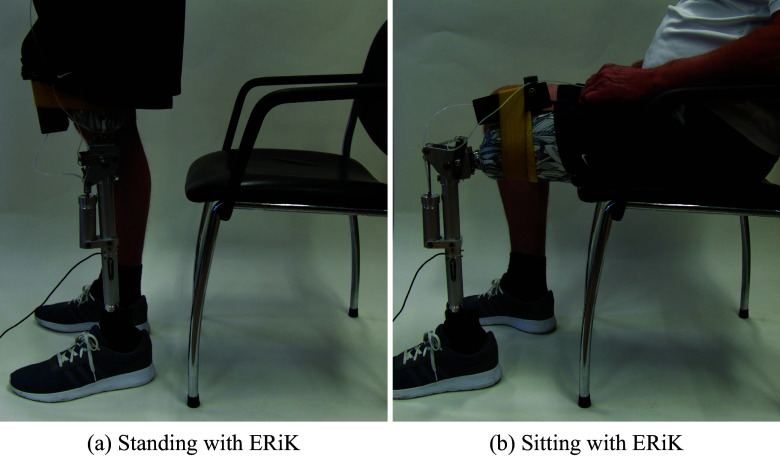
Impression of the Energy Restoring Intelligent Knee prosthesis in standing and sitting configurations. The black box on the thigh contains the valves and manual switch in this photo. The valves and tubing were integrated into the shank prior to the experiments with human participants.

The main component of the system is a pneumatic cylinder (Figure 1a) that connects the upper and lower leg. Figure 1b shows the pneumatic scheme of the system while Figure 2 shows an amputee wearing the Energy Restoring Intelligent Knee (ERiK) in standing and seated positions.

During sitting down, the moving cylinder compresses the air in the cylinder (4). The increased pressure opens the one-way valve (1) and the compressed air flows into a small vessel (6) or accumulator with an initial pre-pressure. The user can now stay seated, while the one-way valve prevents the air from flowing back into the cylinder. In this configuration, the user can freely extend and flex the knee without any resistance as the piston rod can freely slide through the piston. Whenever the user wants to stand up, the cylinder can be loaded again by opening a solenoid pneumatic valve (2). In this way, the pressurized air flows back into the cylinder through the solenoid valve. While standing, the leg is locked in the fully extended position.

The system is a closed one in which air is compressed and decompressed. No fluid streams into or out from the system. In principle, the system is leak tight, meaning that the pre-pressure will remain constant. This is similar to the gas springs used in cars boots and adjustable office chairs, which have a typical leakage of about 1–3% per year.

### 2.2. Design parameter choices

In order to provide substantial support to the sound knee during initiating SU, the prosthesis should be able to deliver a maximum torque in the same order of magnitude as a sound knee of an elderly person. Elderly persons minimally need a total knee torque of 99 N m to initiate SU, which translates to 49.5 N m per knee (Schultz et al., 1992).

The torque the prosthesis provides is determined by the actuator force and the transmission ratio, with several constraints limiting the values of these parameters. In order to increase the maximum knee torque, the actuator diameter and hence the piston surface can be increased, as well as the lever arm of the actuator with respect to the knee joint. The dimensions are, however, also constrained by the outer contours of a human leg as the prosthesis must fit inside normal clothing.

The cylinder diameter was set to 30 mm so that the cylinder would fit next to a standard prosthesis tube. The lever length is closely related to this cylinder diameter because the lever connects the piston rod, which is at the center of the cylinder, to the knee joint, which is located above the center of the prosthesis tube. The lever must be long enough such that the cylinder and the prosthesis tube will not collide during lever motion.

Another parameter that influences the maximum achievable torque is the air pressure. The maximum system pressure is constrained by the limits of the pneumatic components; most standard pneumatic components cannot withstand pressures above 1.6 MPa, which was therefore set as the maximum system pressure. For the storage vessel, we used a CO_2_ canister, which normally stores CO_2_ at a pressure of around 6 MPa at 22°C and has a burst pressure of about 52 MPa, which is much higher than the pressures we used.

A simulation was performed with the chosen parameters in order to check whether the system could generate a substantial knee torque (Lachmann, 2016). For a pre-pressure of 0.5 MPa, the system can generate a peak torque of 30 N m. While this is less than the above-specified value of 49.5 N m, we still expected a noticeable benefit from the system.

The total mass of the prosthetic leg with pyramid connector is approximately 2 kg. A further description of the system can also be found in the patent application filed by Smit and Vallery (2016).

### 2.3. Control

The solenoid valve is designed to be controllable by a microprocessor, which opens and closes the valve based on input from a knee angle sensor and a load sensor, hence the name of the device: the “ERiK.” The focus of the current article is not, however, the detection algorithm, and in these trials, an experimenter triggered the valve manually using an extension cord.

## 3. Technical evaluation: Methods

To validate the theoretical calculations made during the design, the predicted knee torque was compared to the actually delivered knee torque as measured in a table-top setup. The lower leg was mounted to a table and a force was applied to the upper leg. In total, three different settings of the pre-pressure in Chamber A were investigated. They were set prior to testing with a tolerance of ±0.01 MPa measured by an integrated pressure gauge (Type MA-27-16-R1/8, FESTO, Esslingen, Germany).

To quantify torque as a function of the angle, we varied the knee angle starting from *α* = 90° to 0° with a step size of 5°. The solenoid valve was kept open during the entire measurement process. The knee angle was measured using a rotary encoder (AS5048A, ams AG, Premstätten, Austria).

The force was measured by means of a mechanical force gauge (Type LG-100 N 100 N × 0.5 N, Chatillon, NY). All torques were computed as the measured force multiplied by the length of the lever from the center of the knee joint to the contact point of the force gauge (40 cm). Maximum and minimum torque values were recorded for each angle increment in the protocol. The maximum values correspond to a change in direction of an increasing knee angle, whereas the minimum values correspond to a change in direction of a decreasing knee angle.

## 4. Technical evaluation: Results

**Figure 3.**
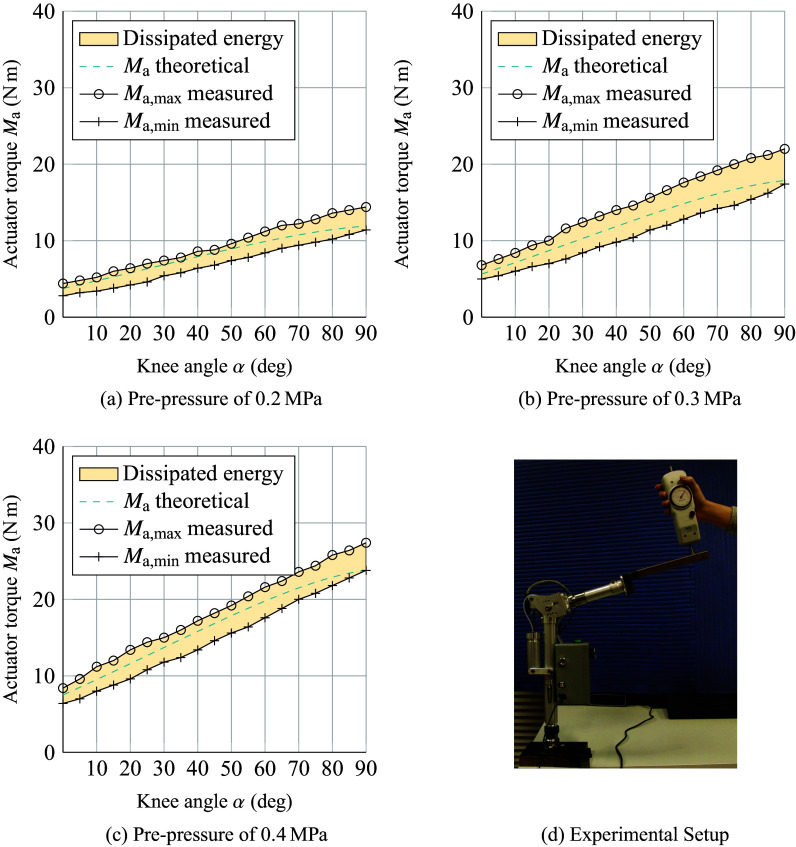
(a–c) Knee torque measured at different knee angles at a prepressure of 0.2, 0.3, and 0.4 MPa, respectively. The dashed lines represent the calculated theoretical pressure based on the pneumatic circuit’s volumes and the set pre-pressure. The enclosed area represents the hysteresis of the cycle of flexion and extension. (d) Experimental setup used to measure angle and force.

Figure 3 shows the results of the torque measurements at given knee angles for pre-pressure settings between 0.2 and 0.4 MPa in comparison to the theoretical values. The markers of the minimum and maximum curves represent the measurement points. The enclosed colored area represents the hysteresis of the system, for example due to friction.

## 5. Pilot study with prosthesis users: Methods

### 5.1. Study goal and approval

This pilot study quantified the effect of support of ERiK on individuals with transfemoral amputation and low activity levels during SU and SD movements. The experiment was approved by the medical ethical review board of Erasmus MC, Rotterdam (NL62211.078.17).

### 5.2. Participants

Only participants who had been fitted with a prosthesis for longer than 1 year and did not have any balance disorders were included in the study. Seven individuals with a transfemoral amputation took part in this pilot study; Table 1 provides an overview of their characteristics. Participant 7 had an osseointegrated transfemoral attachment, while all other participants had conventional sockets.

**Table 1.** Seven participants participated in the study

Participant	Amputation	Sex	Age	Weight incl. prosthesis (kg)
1	Right	Female	64	87
2	Left	Male	59	80
3	Left	Male	79	63
4	Right	Female	59	61
5	Left	Female	73	76
6	Left	Male	73	60
7	Left	Female	52	61

*Note.* All participants had a transfemoral amputation.

### 5.3. Experimental setup and protocol

After the participants had provided informed consent, the ERiK prosthesis was fitted on the residual limb. The pre-pressure of the pneumatic system was adjusted to each participant according to weight and personal preference during initial familiarization trials. The prosthetic leg was fitted with a prosthetic foot (1C30 Trias, 1C30 = N25-1-P/0, OttoBock, Duderstadt, Germany) with a cosmetic covering, corresponding to the side of amputation.

To characterize each individual’s motor abilities, we used the Short Physical Performance Battery (SPPB) protocol. The SPPB is a standardized protocol developed by the National Institute on Aging to objectively assess lower limb function and mobility in elderly people (Guralnik et al., 1994). The SPPB comprises three parts: the “Balance Test,” the “Gait Speed Test,” and the “Chair Stand Test.” The participants were asked to perform the tests while wearing the ERiK prosthesis as indicated in the SPPB protocol. During the Chair Stand Test, the participants were seated on a piano stool that had no armrest. The stool height was adjusted such that the knee angle was approximately 90° when seated.

To evaluate the support function of ERiK, we modified the SPPB slightly, focusing on the task “Repeated Chair Stand Test” (RCST), which is part of the “Chair Stand Test” and for which the participant is to make five sit-to-stand transitions as quickly as possible. In this pilot study, the RCST was performed twice, once without knee support and once with active knee support in order to compare both conditions.

The participants were randomly assigned to one of two groups: one in which the support condition was tested first and one where the unsupported condition was tested first. This was done to prevent potential learning from affecting the results. One experimenter remotely enabled and disabled the support function of the prosthesis depending on the experimental condition. In between both RCSTs, participants were given a 15 min recovery break.

Contrary to the original SPPB protocol, the participants were not required to cross their arms in front of their chest during the Chair Stand Test. Participants had to hold the knee-unlocking cable with one hand to unlock the extended knee before sitting down. We asked participants not to use their hands while standing up. They were, however, allowed to do so whenever they felt it to be necessary. To allow comparison of both RCST conditions, the completion of the task was considered to be more important than the exact execution of the original RCST protocol.

Participants had to make a full stand-up each time, which means their legs should be fully extended, and they had to fully sit down. We verified this using the force plate beneath the piano stool.

Before the main data collection, participants were allowed to familiarize themselves with the prosthesis and its support function by slowly performing a few SU and SD movements. However, the number of stand-ups was constrained in order to avoid fatigue.

Three-dimensional kinematic data were acquired by a motion capture system (Qualisys AB, Gothenburg, Sweden) comprising 12 cameras and markers placed over bony landmarks, following the markerset described in Leardini et al. (2007). Ground reaction forces (GRFs) were captured by three force plates (Kistler, Winterthur, Switzerland), one under each foot and a larger one placed under the stool.

### 5.4. Data processing and outcome measures

The primary outcome measure was the time taken to stand up five times, as performed during the RCST. We hypothesized that support from the ERiK device would reduce the duration. As a secondary outcome measure, the weight-bearing ratio during transitions was calculated by first dividing the vertical GRF on the prosthetic limb by the combined GRF of both legs, and then taking the median of this ratio for all transition samples in a trial. A value of 1 indicates that all weight is on the prosthetic leg and a value of 0 means all the weight is placed on the sound leg. We hypothesized that with support, participants would place more weight on the prosthetic leg during the transitions.

Kruskal–Wallis nonparametric tests were conducted to compare supported and unsupported conditions in terms of primary and secondary outcome measures, with the significance level set at *α* = 0.05. This nonparametric test was chosen because of the low number of participants.

To obtain more insight into the participants’ movement strategies, we also calculated joint angles and moments from the motion capture and force plate data. Missing marker positions were estimated using a combination of linear interpolation over time and the method introduced by Gløersen and Federolf (2016). Joint angles were extracted from the marker positions using custom MATLAB (Mathworks, Natick, MA) code.

The sound and prosthetic knee torques were each calculated from the GRF vector at the foot and its location with respect to the knee joint. This simplified calculation neglects inertial and gravitational effects of the shank, but those effects are small with respect to the knee moment generated by the ground reaction force. We verified this assumption using full inverse dynamics.

Individual SU and SD movements were obtained from the data by identifying start and stop times based on kinematic data from the sound and prosthetic legs. This provided the times taken to perform the individual movements, allowing comparison between the support and no support trial conditions. Furthermore, the work done by the sound and prosthetic legs during SU and SD could be evaluated by taking the integral of the torque-angle curve (via the trapezoidal rule); the mean work done by a participant during standing up and sitting down could then be calculated from these data.

## 6. Pilot study: Results

### 6.1. SPPB results

Table 2 shows the results of the SPPB test while participants were wearing the ERiK prosthesis (without active support). Participants achieved a total score of 6.0 ± 2.5 on the SPPB. This is a relatively low score. For comparison, healthy individuals with two anatomical legs can reach a maximum score of 12 points. Participants with a transfemoral amputation (average age: 43.3 years) in a study by Hoffman et al. (2002) scored 9.3 ± 2.7. The lower SPPB score in our study reflects our aim to include less mobile, older participants (average age: 65.6 ± 9.0 years). Moreover, the ERiK leg prototype, can only walk with a stiff knee. This has a negative effect on the SPPB score.

**Table 2.** Scores of the SPPB test while using the ERiK prosthesis without support

Participant	Balance test	Gait speed test	Chair stand test	Total score
1	3	1	0	4
2	4	4	1	9
3	1	2	0	3
4	3	2	1	6
5	2	1	0	3
6	4	3	1	8
7	4	4	1	9

*Note.* The SPPB consists of four parts for which one can score four points, yielding a maximum score of 12 points for participants in good physical shape.

As explained in the previous section, all participants were allowed to provide push-off with their hands against the stool during stand-up. This is not allowed in the standard SPPB and would yield a score of 0 points for the Chair Stand Test.

### 6.2. Qualitative observations

We noticed that the pre-pressure that could be set was rather low for all participants (around 0.2–0.3 MPa), predicting also that the expected support would be very limited. The main reason for this setting was that with higher pressure, it became more challenging for participants to initiate standing up and to control the leg. The participants, therefore, preferred a lower pre-pressure setting so that it would be easier for them to initiate sitting down and to control the leg during standing up and sitting down. Therefore, although the leg could provide considerably more torque, we tested the leg at a lower pre-pressure and thus lower torque.

**Figure 4.**
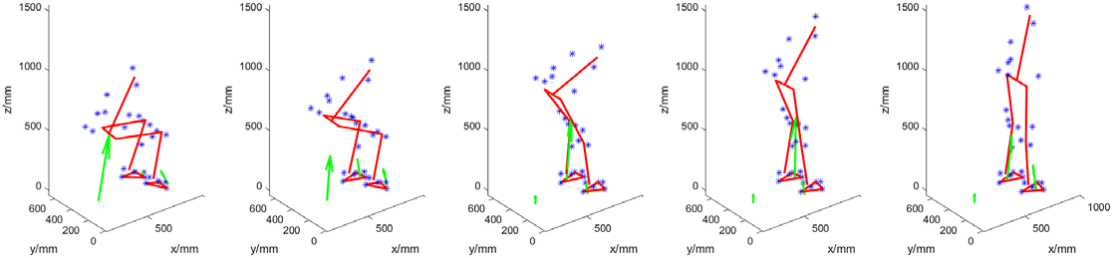
Participant 4 standing up with support. Initially, the participant places the prosthetic foot (right) on the toe, as is also visible by the location of the COP at the front of the foot. This helped prevent the foot from slipping due to knee torque. The main weight during the transition remains on the sound (left) leg. Throughout the transition, the upper body leans forward.

The SU movement was characterized by an initial position with the prosthetic foot placed in front of the sound foot in all participants. The prosthetic foot was often positioned on the toe to achieve some degree of dorsiflexion. Nevertheless, the rigidity of the foot appeared to be a problem because the ankle remained at an approximate angle of 90°, complicating appropriate placement to initiate standing up. An exemplary initial position and full SU transition (of Participant 4) are shown in Figure 4.

With support enabled, the prosthetic knee tended to be fully extended before the sound knee was and before trunk extension commenced. In some familiarization trials, this premature extension resulted in balance issues for the participant. Participants also started SD with the prosthetic foot placed in front of the sound foot.

In some trials, both with support and without, participants had difficulties relating to getting the prosthetic knee to flex from its locked position. This appeared to be due to a lack of available hip torque to force the leg to buckle out of its singular configuration. In those trials, the participants started by flexing their sound knee, with insufficient weight being placed on the prosthesis. This caused the COP to move toward the heel and the GRF line of action to pass in front of the knee, leading to an extension moment about the knee. Generally, prosthetic knee flexion occurred only after the prosthetic foot was placed on the toe and the residual limb was pressed forward to effect a flexing torque. After knee flexion, sitting down was most accurately described as controlled collapse.

In a few cases, the leg suddenly extended once the participant was seated. This happened if the knee was not fully flexed during SD, such that pressurized air remained in the cylinder. This residual pressure was sufficient to cause the lightweight leg to extend even though the valve (2 in Figure 1b) was closed.

**Figure 5.**
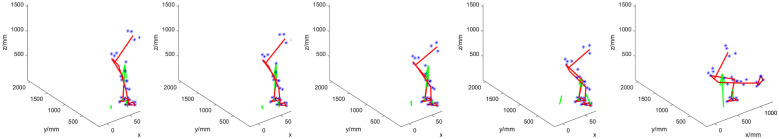
Worst-case Sitting Down: Participant 1 performing this transition with support. She is firstly struggling to initiate knee flexion. The trunk is flexed as well as the sound leg. However, the participant is unable to overcome the pre-tension extension torque of the prosthesis. Lacking sufficient hip torque and the possibility of transmitting this torque via the prosthetic shaft, she instead tries to adjust her posture and thereby ground reaction force to move the knee out from its singular position. Throughout the transition, little weight is on the prosthesis (right leg). At the end of the transition, the leg also suddenly extends because the full bending angle had not been reached, so the cylinder was still pressurized while there was no weight on the foot. The participant then needs to manually bring the leg back down, which takes time.

Figure 5 illustrates a worst case, namely when Participant 1 struggled to sit down. The participant firstly had difficulty in initiating knee flexion, and at the end of the transition could not control the foot on the ground due to a lack of weight on the leg, such that the knee did not fully flex, residual air was left in the cylinder, and the leg was extended as a result. Due to the low weight of the prosthesis, a small amount of residual air is sufficient to move it upwards.

Overall, the support of the leg did not appear as a substantial help to the participants, and they required several attempts to learn how to use the system. Nevertheless, several participants told us that they had the impression that the leg was helping them perform the movements when it provided assistance.

### 6.3. Quantitative outcome measures

Contradicting our hypothesis for the primary outcome measure, each individual took longer to stand up five times with support than without. The Kruskal–Wallis test did not show significance between conditions (*p* = .055), but Table 3 shows a trend in the individual times.

**Table 3.** Time taken to stand up five times (and to sit down four times in-between)

Participant	Without support (s)	With support (s)
1	64.0	103.0
2	25.0	47.8
3	55.6	72.9
4	27.8	53.0
5	32.4	65.9
6	22.7	27.8
7	26.3	38.0

**Figure 6.**
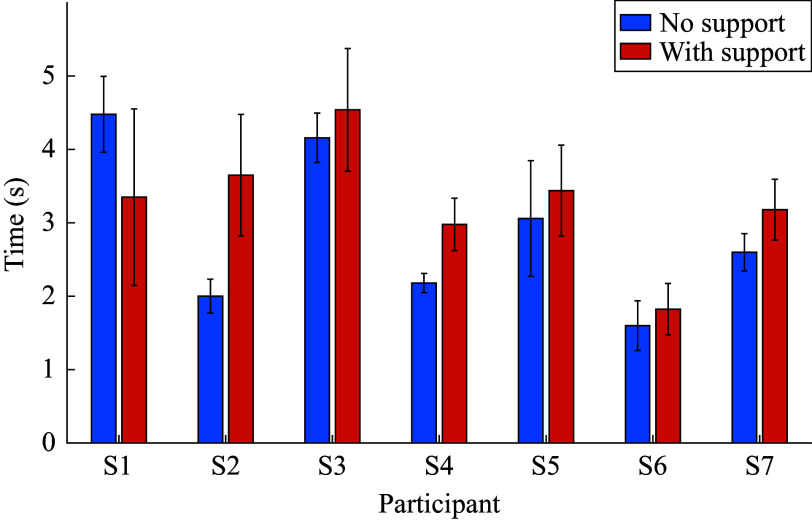
Average stand-up times with standard deviations represented by error bars for each participant for no support and support conditions.

**Figure 7.**
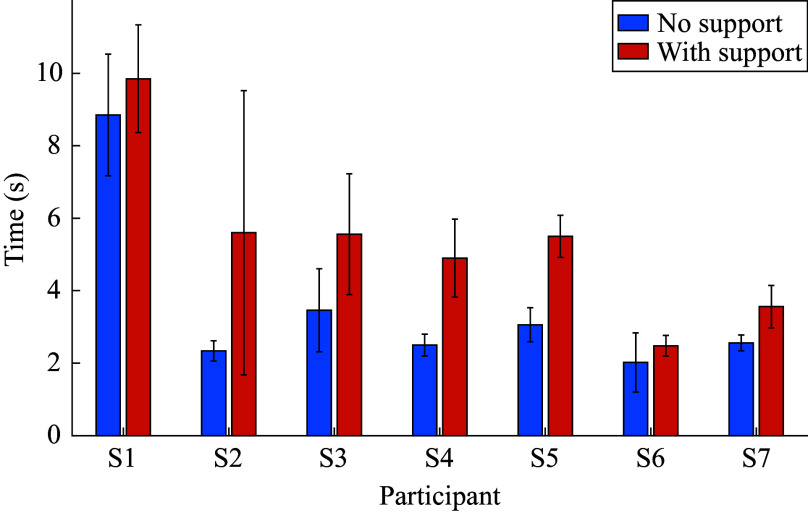
Average sit-down times with standard deviations represented by error bars for each participant for no support and support conditions.

Figures 6 and 7 illustrate the mean times taken for each participant for standing up and sitting down, respectively. In these calculations, only the time from the start of each stand-up or sit-down till the end of that individual movement is included, with the transition time between each movement, which represents the “initiation” of the stand-up or sit-down movement – being excluded. The figures again show that the time needed for the various participants to complete the given movement tends to be higher when the support is provided.

For the secondary outcome, Table 4 reports the fraction of load supported by the prosthetic leg. The table shows the median load bearing of the prosthetic side for the seven participants during transitions as a percentage of total load on the feet. The weight distribution of all participants was asymmetric toward the sound side, with little weight being put on the prosthesis, regardless of the condition.

**Table 4.** The median percentage of prosthetic-side weight bearing during transition phases, calculated as the vertical GRF of the prosthetic side divided by the total GRF on the feet

Participant	Without support (%)	With support (%)
1	13.8	19.8
2	12.1	17.3
3	11.6	16.6
4	23.2	24.7
5	12.2	17.9
6	21.9	27.3
7	23.0	25.9

The Kruskal–Wallis test did not confirm a significant group effect between the two conditions (*p* = .11).

Table 5 shows the mean work done by the knee joint for the sound and prosthetic legs during the standing-up phase. The data show that the work done by the sound leg tended to be slightly smaller when the support was provided, while the prosthetic leg, as expected, performed little work during the no support condition (some values are negative as a calculation artifact due to errors relating to the inverse kinematics) but a greater degree of work during support, though still lower than that of the sound leg.

**Table 5.** Mean work done by the sound and prosthetic (pros) legs during standing up for each participant for no support (NS) and support (S) conditions

Participant	Sound, NS (J)	Sound, S (J)	Pros, NS (J)	Pros, S (J)
1	50.2	36.3	0.38	21.1
2	97.9	81.3	2.49	14.5
3	35.4	32.3	−0.070	12.4
4	31.9	22.6	0.190	11.7
5	64.0	57.7	−1.13	12.9
6	72.2	63.3	0.923	15.7
7	64.9	55.5	−1.48	10.6

Corresponding mean work done data for the sitting down movement are shown in Table 6. The evaluated work done values here are negative as the moments are applied in the opposite sense to the angular velocities. Again, the work done by the sound leg tends to be smaller when the support is provided, while the prosthetic leg does little work in the no support condition but does provide more resistance during the support condition.

**Table 6.** Mean work done by the sound and prosthetic (pros) legs during sitting down for each participant for no support (NS) and support (S) conditions

Participant	Sound, NS (J)	Sound, S (J)	Pros, NS (J)	Pros, S (J)
1	−50.4	−25.5	−1.42	−32.6
2	−89.7	−73.3	−8.26	−26.9
3	−55.1	−46.9	−3.38	−20.9
4	−43.8	−28.9	−4.97	−22.1
5	−60.6	−57.7	−3.52	−23.6
6	−70.1	−60.0	−3.07	−24.5
7	−74.7	−57.7	−1.21	−18.5

**Figure 8.**
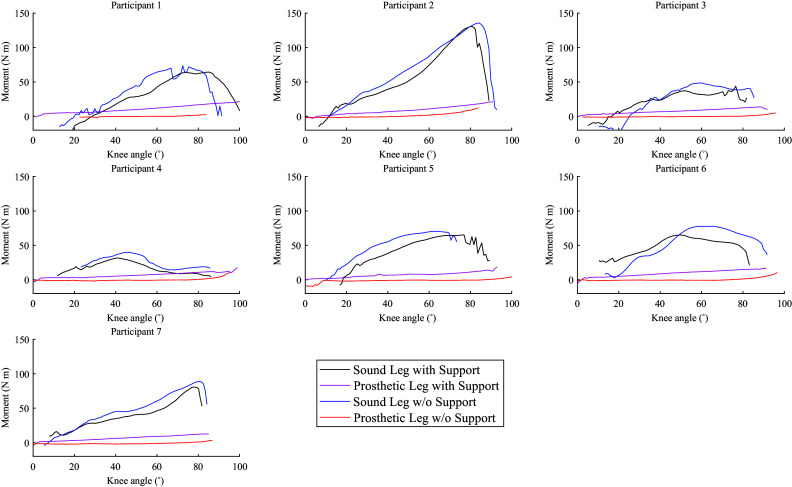
Mean knee moments of prosthetic and sound legs plotted versus knee angle for each participant during standing up.

Figure 8 shows the average knee moments, calculated via inverse dynamics for the prosthetic and sound legs for the standing transition. Each curve represents mean across the individual moment-angle curves during standing for each participant. Furthermore, in the calculation, weight and inertia of shank and foot were neglected.

**Figure 9.**
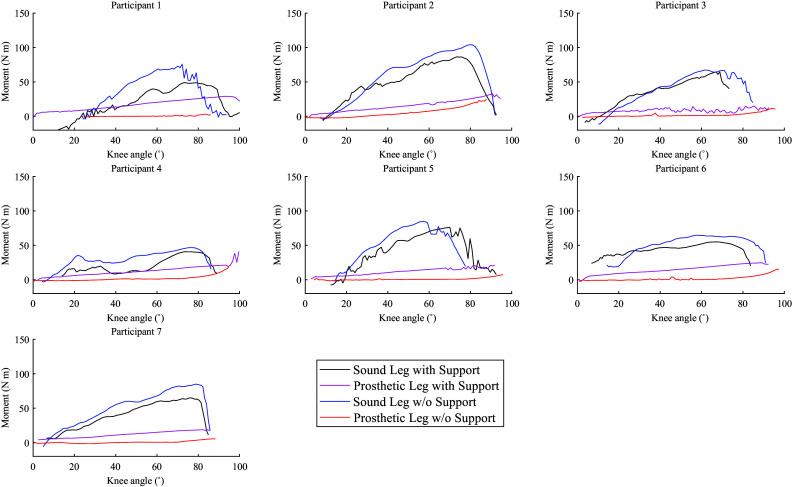
Knee moments of prosthetic and sound legs plotted versus knee angle for each participant during sitting down.

The average knee moments for the prosthetic and sound legs during sitting down are shown in Figure 9. In common with the data for the SU movements, the moments produced by the sound leg tended to be slightly lower when support is provided. The moment observed for the prosthetic leg appears low during the no support condition but shows higher values during the support condition trials.

## 7. Discussion

### 7.1. Functionality of the current prototype

Figure 3 shows that the system is able to regenerate a considerable portion of the input torque, which means that losses due to storing and releasing the potential energy are low. However, the results from the evaluation study indicate that there is little functional benefit of the support during both SU and SD movements.

As shown in Table 4, the median portion of weight supported by the prosthetic side during transitions increased for each of the seven participants in the condition where the prosthesis supported the movement. Hence, the distribution of the participants’ weight over the two limbs tended to be slightly more symmetric with support enabled. However, this improvement is relatively small, not statistically significant, and no participant came close to 50% weight bearing on the prosthetic side, meaning the sound leg was not substantially unloaded.

Figures 8 and 9 help illustrate this observation using joint torques: even though a trend is visible for sound-side knee torque to slightly reduce when support is enabled, it is also apparent that the knee torque provided by the pneumatic actuator is too low to generate substantial relief. The torques of the sound leg are generally a little lower than those reported in Burger et al. (2005) in both conditions, but this is consistent with the difference in body weights.

The experiments confirmed that the knee torque of the sound leg can indeed be well-approximated as a function of the angle (Figures 8 and 9), regardless of the direction in which the person is moving. This justifies the general choice of a design with an angle-dependent torque. The figures also show that the prosthetic knee torque is an almost linear function of the angle, as expected from the current design and consistent with the table-top experiments (Figure 3).

The lack of ankle function was a major drawback of the design and may explain why most participants started both transitions on the toe of the prosthesis. Positioning on the toe achieves a similar effect as ankle dorsiflexion: namely, to maintain the center of mass over the base of support. Positioning the prosthesis on the toe when initiating SU also prevents it from slipping forwards, as a knee extension torque pushes the toe into the ground. This is a benefit over placing the foot flat on the ground because in that case, the foot might start slipping forward if the knee extension torque exceeds the maximum possible friction force between foot sole and ground surface.

Training was outside the scope of this study; given the physical condition of participants, they would have easily fatigued when asked to stand up many times. However, we already observed some degree of learning during the few familiarization movements: in their initial attempts to use the system, participants seemed reluctant to put weight on the prosthesis, possibly due to a lack of confidence in its support. They also initially struggled with flexing the prosthetic knee during SD, and with a prematurely extended knee during SU. This improved in later trials for most participants. This observation suggests that training would be necessary to make full use of the system and improve performance. However, improvements to the design and performance of the prosthesis device would be beneficial before embarking on a further study focused on training.

### 7.2. Future work

The kinematic issues with the device might be solved by adding a degree of freedom in the ankle joint and through increasing the knee range of motion. These adjustments would allow participants to place the prosthetic foot further back, thereby reducing the distance the body’s center of mass has to travel during SU. Furthermore, they would enable participants to have a more natural and stable descent during SD.

In principle, a higher pre-pressure could help increase the amount of support provided by the prosthesis. However, with the current design and binary method of control, this is not feasible, since in the current design, there is a considerable residual force during quiet sitting and quiet standing. During sitting, this might lead to unwanted knee extension. During standing, this leads to difficulties in flexing the knee and thereby impedes sitting down.

A first option to enable higher pre-pressures would be to employ a more gradual control method, also taking into account the load on the leg and avoiding binary switching. Control methods already exist that are based on user intent, for example by Simon et al. (2016). This, however, requires a proportional valve, which would also increase the complexity of the system. A second option would be to adjust the nonlinear transmission between actuator force and knee torque by reducing the mechanical lever arm further around the configuration where the knee is extended. This could specifically facilitate SD.

## 8. Conclusion

While the device was theoretically able to allow pre-pressures of up to 0.5 MPa, prosthesis users requested much lower pre-pressures in practice due to the foot slippage. Consequently, with the lower chosen pre-pressures, the device provided a correspondingly lower supporting knee torque than its maximum capability. In this way, it did not provide a tangible functional benefit to users, meaning that the time taken to perform the movements did not decrease and, furthermore, that the sound leg was only marginally relieved. Moreover, a main observation from the study was that the rigid ankle hindered the participants in exploiting the knee support.

The lessons learned during this study may help guide future designs. While lightweight energy-recovering prostheses such as ERiK have the potential to help elderly amputees maintain or regain their mobility, an ankle joint that can dorsiflex appropriately, as well as the incorporation of a less binary control method, are likely needed to simplify movement initiation.

## Data Availability

The data supporting the technical evaluation and the derived data supporting the findings of the pilot study with the participants, together with the custom Matlab scripts to process the data, are publicly available via the 4TU repository. Due to privacy restrictions, raw data of the pilot study have not been made available.
